# Dynamical Organization of Syntaxin-1A at the Presynaptic Active Zone

**DOI:** 10.1371/journal.pcbi.1004407

**Published:** 2015-09-14

**Authors:** Alexander Ullrich, Mathias A. Böhme, Johannes Schöneberg, Harald Depner, Stephan J. Sigrist, Frank Noé

**Affiliations:** 1 Department of Mathematics, Freie Universität Berlin, Berlin, Germany; 2 Institute for Biology/Genetics, Freie Universität Berlin, Berlin, Germany; 3 NeuroCure Cluster of Excellence, Charité Berlin, Berlin, Germany; The Krasnow Institute for Advanced Studies, UNITED STATES

## Abstract

Synaptic vesicle fusion is mediated by SNARE proteins forming in between synaptic vesicle (v-SNARE) and plasma membrane (t-SNARE), one of which is Syntaxin-1A. Although exocytosis mainly occurs at active zones, Syntaxin-1A appears to cover the entire neuronal membrane. By using STED super-resolution light microscopy and image analysis of *Drosophila* neuro-muscular junctions, we show that Syntaxin-1A clusters are more abundant and have an increased size at active zones. A computational particle-based model of syntaxin cluster formation and dynamics is developed. The model is parametrized to reproduce Syntaxin cluster-size distributions found by STED analysis, and successfully reproduces existing FRAP results. The model shows that the neuronal membrane is adjusted in a way to strike a balance between having most syntaxins stored in large clusters, while still keeping a mobile fraction of syntaxins free or in small clusters that can efficiently search the membrane or be traded between clusters. This balance is subtle and can be shifted toward almost no clustering and almost complete clustering by modifying the syntaxin interaction energy on the order of only 1 k_B_T. This capability appears to be exploited at active zones. The larger active-zone syntaxin clusters are more stable and provide regions of high docking and fusion capability, whereas the smaller clusters outside may serve as flexible reserve pool or sites of spontaneous ectopic release.

## Author Summary

For the communication between two nerve cells, a synaptic vesicle containing neurotransmitters has to fuse with the neuronal membrane at a specific fusion site, releasing its signaling molecules. The vesicle fusion is mediated by a specific family of proteins (SNAREs) that are located on the vesicle as well as on the neuronal membrane. As many other membrane proteins, SNARE proteins are not uniformly distributed over the membrane but rather exist in complexes or clusters. Syntaxin, one of the SNARE proteins, is known to form such clusters through attractive protein-protein interactions. With the help of light microscopy techniques we show that the actual size and abundance of these clusters depends on its proximity to the fusion site on the membrane. We developed a computational model of Syntaxin cluster formation that can explain the observed differences in clustering and allow us to speculate on their potential role in the process of docking and fusion of synaptic vesicles. The formation of clusters through weak protein-protein interactions allow for a highly dynamic behavior of proteins, being able to easily switch between a state with stable and almost immobile clusters and a more dynamic situation with clusters exchanging particles at high rates.

## Introduction

Synaptic exocytosis occurs efficiently at active zones, where neurotransmitter containing synaptic vesicles (SV) are docked, primed, and released [1]. Synaptic vesicle fusion is mediated by complex formation between three members of the family of soluble N-ethyl-maleimide-sensitive fusion protein (NSF) Attachment protein Receptor (SNARE) proteins, Syntaxin-1A and SNAP-25 on the presynaptic membrane (t-SNAREs) and Synaptobrevin on the vesicular membrane (v-SNARE) [2,3]. Syntaxin-1A and SNAP-25 are known to form microdomains [4,5]. In this context, super-resolution microscopy studies have shown that Syntaxin-1A clusters are about 60 nm wide and have been estimated to contain around 75 Syntaxin-1A molecules [6]. Various hypotheses for the mechanism of cluster formation are discussed, including weak homophilic protein-protein interactions [5,7], interactions with other membrane or scaffold proteins [8–10], or specific membrane components such as cholesterol or phosphoinositides [4,11–15]. Similarly, different roles of SNARE clusters in the docking, priming and fusion process of vesicles have been proposed but are still under debate [16–23].

Although SNAREs catalyze exocytosis, which predominantly occurs at active zones, SNARE clusters are found all over the neuronal membrane. Previous studies have not provided evidence that SNARE proteins or microdomains would be preferentially located at active zones [24–29], as confocal images show only slight differences in intensities between synaptic and extrasynaptic regions [30]. A multitude of super-resolution microscopy studies have been performed on PC-12 cells that do not possess active zones. For exocytosis of insulin containing granulae, TIRF microscopy has revealed that SNAREs co-localize with granules [11,18,19]. Moreover, single molecule as well as FRAP experiments at rat spinal cord neurons [30] showed a change in the mobility of Syntaxin-1A depending on its localization with respect to active zones. Syntaxin-1A molecules at active zones reveal a slower and more confined diffusion with more pauses. Ribrault et al suggested these changes in dynamics to be caused by changed association rate of SNARE complexes, but stated that clusters of SNARE proteins could also account for this observation.

Here we use Stimulated Emission Depletion (STED) super-resolution light microscopy [31] to investigate differences in the distribution and sizes of Syntaxin-1A clusters between active zones and other regions of the neuronal membrane, in fixed larvae at *Drosophila* neuromuscular junctions (NMJs). Diffusion simulations [32] were used to relate Syntaxin-1A dynamics and clustering. Based on this spatiotemporal model, the relevance of syntaxin-1A clustering for the efficiency and sustainability of exocytosis is discussed.

## Materials and Methods

### Antibody generation

For a polyclonal Bruchpilot-C-terminal antibody, the coding sequence corresponding to the last 200 amino acids of the 190 kDa isoform (aa 1541–1740 [33,34]) was amplified from the bruchpilot cDNA [33] and cloned into pGEX-6P-1 and pET28ausing BamHI and XhoI restriction sites (forward PCR primer: GTACGGATCCCAGCTGCAGCAGCAGATGCAACAA; reverse PCR primer: GTCTCTCGAGTCAGAAAAAGCTCTTCAAGAAGCCAGC. The purified GST fusion protein (GST-BRP-last200) was injected into a rabbit and the antiserum was affinity purified with the 6xHis-BRP-last200 fusion protein.

### Immunostainings for STED microscopy

Dissections and immunostainings were essentially performed as described previously [35]. But briefly as follows: Midstage third-instar larvae were put on a dissection plate with both ends fixed by fine pins and then covered by a drop of ice-cold hemolymph-like saline (HL-3) solution. Dissection scissors were used to make a small hole at the dorsal midline of the larva (near to the posterior end), which was then completely opened along the dorsal midline from the hole to the anterior end. The epidermis was stretched flat and pinned down, and then the internal organs and CNS were removed carefully with forceps, and then the epidermis was stretched flat and pinned down. The dissected samples were fixed and then incubated with primary antibodies overnight, followed by fluorescence-labeled secondary antibodies and mounted in Mowiol (Max-Planck Institut for Biophysical Chemistry, Group of Stefan Hell). The genotype w1118 was used and all larvae were raised at 25°C on semi-defined medium (Bloomington recipe). All fixations were performed for 10 min with 4% paraformaldehyde (PFA) in 0.1 mM phosphate buffered saline (PBS). Primary antibodies were used in the following concentrations: rabbit polyclonal anti-BRP^C-term^ (Last200, this study) 1:1000 and mouse monoclonal anti-Syntaxin (8C3) 1:50 (Developmental Studies Hybridoma Bank, University of Iowa, Iowa City). Secondary antibodies for STED were used in the following concentrations: goat anti-mouse Atto590 1:100 and goat anti-rabbit star635 1:100 (Atto590 (ATTO-TEC) and star635 (Abberior) coupled to respective IgGs (Dianova)). Images from fixed samples were taken from 3rd instar larval NMJs.

### STED microscopy

STED microscopy was performed as previously described in [36,37]. Briefly: Two-color STED images were recorded on a custom-built STED-microscope [38], which combines two pairs of excitation laser beams of 595 nm and 640 nm wavelength with one STED fiber laser beam at 775 nm and achieves a lateral resolution of approx. 40 nm. STED images were processed using the linear deconvolution algorithm integrated into Imspector Software (Max Planck Innovation GmbH). The point spread function (PSF) for deconvolution was estimated to be a 2D Lorentz function with a full width at half maximum fitted of 25nm. Images were processed with ImageJ software to remove background.

### Image analysis

Image analysis was programmed in Matlab and performed on the deconvolved data. Although the deconvolution algorithm affects the apparent cluster size, the relative size is maintained. To identify clusters of Syntaxin-1A and spots of Bruchpilot, the local maxima in the intensity profile of the respective channel of the STED images are sought. For this, a circular window of fixed diameter (9 pixel for syntaxin-1A, 21 pixel for Bruchpilot, 1 pixel = 10nm) is shifted over all pixels of an image (1500x1500 pixels) and it is tested whether the current pixel is the maximum value within the window. Local maximum pixels with intensities >25 a.u. are considered to be cluster centers. Thus, clusters can be distinguished when they are more than 40nm apart (for Syntaxin) and 100 nm apart (for Bruchpilot).

To determine the size of the Syntaxin-1A clusters, the full-width-half-maxima area is defined as the cluster area. Starting from the cluster center, equidistant pixels are scanned until a distance is reached at which the average pixel intensity drops below half the intensity of the cluster center. All scanned pixels with an intensity above or equal to the half-maximum are considered to belong to the cluster area. The diameter of the cluster is defined as the diameter of a circular area of the same size as the cluster area. Binding of the secondary to the primary antibody may increase the distance of the fluorophore from the used epitope and therefore lead to an overestimation of the calculated absolute cluster size. However, the comparison of cluster sizes is still valid.

**Fig 1:**
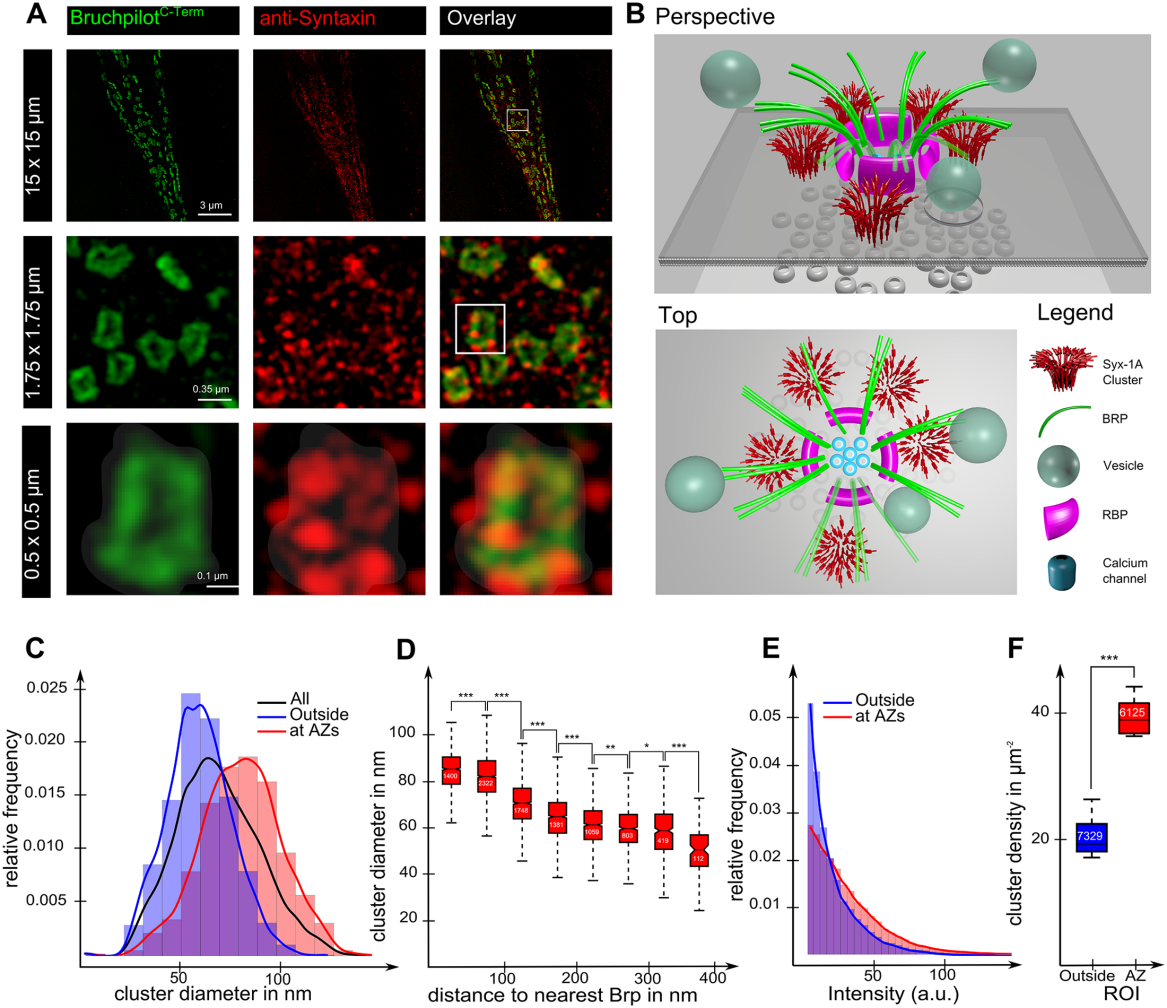
Syntaxin 1A forms larger clusters at active zones. (A) STED images of a Drosophila neuromuscular junction (NMJ), co-stained for Bruchpilot (left, green) andSyntaxin-1A (middle, red), and their overlay (right). Top row (15 μm x 15 μm): Syntaxin-1A is abundant over the entire NMJ and Bruchpilot forms ring-like structures. The middle row (1.75 μm x 1.75 μm): zoom showing seven active zones indicated by the Bruchpilot rings. Syntaxin-1A appears in patchy structures identifying clusters. Bottom row (0.5 μm x 0.5 μm): zoom showing one Bruchpilot ring. Syntaxin-1A micro-domains situated beneath or near the Bruchpilot ring structure. The active zone region as defined here is shown as shaded region. (B) Illustration of an active zone model showing the Bruchpilot and Syntaxin-1A cluster positions as observed in the STED images. (C) Analysis of the Syntaxin-1A cluster size with respect to their position towards the active zone. The cluster size distribution of identified Syntaxin-1A clusters, with cluster sizes defined by the diameter of the full width half maximum area. The distribution for whole NMJs (All) as well as the distributions in Syntaxin-1A clusters at (at AZ) and outside of active zones (Outside) are shown. (D) Syntaxin cluster size as a function of their distance to the nearest active zone (BRP ring structure). Boxplots show the median and distribution of cluster sizes for 8 distance ranges. Asterisks indicate degree of statistical significance and are inferred from the probability (P-Value) of the difference in means using a T-test, * = P<0.05, ** = P<0.01, *** = P<0.001. Asterisks are attached to bars which indicate the corresponding pair being compared in the T-test. Notches indicate 95% confidence interval for the median. The number of clusters within a specific range is shown inside the boxplot. (E) The fluorescence intensity of Syntaxin (red channel) for active zones (green channel intensity above zero) and outside active zones. (F) The density of clusters at the active zone compared with that outside of active zones. The number of clusters for a specific location is shown inside the boxplot.

An active zone is defined as the region surrounding a ring of Bruchpilot spots, with a 25 nm padding inside and outside of the ring structure, resulting in a closed circular area with a diameter of around 250 to 300 nm (Fig 1A, bottom row). Thus, cluster size distributions for syntaxin-1A molecules at and outside of the active zone can be computed. Furthermore we can compute the density of clusters and also their distance to the closest Bruchpilot structure or spot.

The density of clusters at active zones is computed as the number of active zone clusters in one NMJ divided by the cumulated area of all active zones within this NMJ. For the density of outside clusters we determine an area for the entire membrane and subtract the active zone area. The membrane area is defined as area with increased fluorescence intensity and can be easily identified by eye and is selected manually.

### Particle-based computational model

The computational model is simulated with the particle-based reaction-diffusion simulation software ReaDDy [32,39]. In ReaDDy, molecules are represented individually in continuous space and time and their diffusion is described by Brownian dynamics. All molecules are modeled as spherical particles or groups of such particles. The Brownian dynamics equations of motion for particles are solved numerically using an Euler discretization with a constant timestep. ReaDDy supports particle interaction potentials, such as soft-core repulsion potential, allowing to model space exclusion, molecular crowding and aggregation. The particle motion depends on the gradient of these interaction potentials. In principle, all kinds of pairwise potentials can be defined, however, the shape and steepness of the potential affect the choice of the timestep. Therefore, soft harmonic potentials are preferred over Lennard-Jones type potentials because they allow for the use of larger timesteps. ReaDDy further includes reactions between particles that only occur upon physical encounter of reactive particles. While this feature is not used in the present study, it allows for many biological applications [40] such as signal transduction [41].

Here for Syntaxin-1A a minimalistic model is chosen that permits to model the spatiotemporal behavior relevant to this study while allowing for efficient generation of long time series and thus gain statistically significant data. Each Syntaxin-1A molecule is modeled as a dimer of two connected particles. Particle 1 represents the syntaxin transmembrane helix and SNARE domain of the protein: it acts as membrane anchor and has an attractive interaction potential with other particles of the same type, thus giving rise to clustering. Particle 2 represents the N-terminal domain of syntaxin-1A and serves only to introduce the steric repulsion due to the bulky head domain. The radius of the second particle (***r***_**2**_ = **3.3 *nm***) is chosen to approximate the size of the N-terminal domain, the first particle is somewhat smaller (***r***_**1**_ = **3.0 *nm***). The ratio ***r*2**/***r*1** of the two sizes (here: 1.1) determines the strength of repulsion between two Syntaxin-1A molecules due to steric hindrance and thus strongly influences the average cluster size and more importantly the maximal cluster size. Ratios smaller or equal to 1.0 lead to unbounded cluster growth, while larger ratios tend to decrease cluster sizes dramatically. In our case, a ratio of 1.1 sets a practical cluster size limit at 120–140 nm as observed in STED experiments, while ratios of 1.25 and 1.5 reduce this limit to 80–90 nm and 40–45 nm, respectively The diffusion coefficient of the particles is set such that the dimer model has an effective diffusion coefficient of ***D*_*syx*_** = **0.2** μ***m*^2^*s*^-1^**, as found in single molecule experiments [30].

All interactions of particles with other particles or its surroundings are modeled by potentials acting on the particle. Here we need three types of potentials to describe all interactions. The first type of potential, which we will call a membrane potential because it forces the particle on a two-dimensional surface, is described by the following function.

$$U(d)=\frac{1}{2}k_{m}d^{2}$$

Where ***k***_***m***_ ***= 20k***_***B***_ ***T*** is the force constant of the potential and ***d*** is the distance of particle 1 to the membrane surface. Next, a repulsion potential acts between particles 2–2 and 2–1, and is modeled as the harmonic potential:

$$U(d_{x,y})=\left\{ \begin{array}{l} \frac{1}{2}k_{r}{\left(d_{x,y}-r_{x,y}\right)}^{2}\\ 0 \end{array}\right.\;\begin{array}{l} d_{x,y}<r_{x,y}\\ else. \end{array}$$

Here, ***d***_***x*,*y***_ is the distance between two particles ***x*** and ***y***, ***k***_***r***_ ***= 2 k***_***B***_ ***T***, is the force constant of the repulsion potential, and ***r***_***x*,*y***_ ***= r***_***x***_ ***+ r***_***y***_ is the sum of the two particle radii. The third and most important potential for this simulation setup is the attraction or clustering potential defined in the following functions acting between particles 1–1:

$$U(d_{x,y})=\left\{ \begin{array}{l} \frac{1}{2}k_{r}{\left(d_{x,y}-r_{x,y}\right)}^{2}-E_{a}\\ \frac{2E_{a}}{{\left(i_{x,y}-r_{x,y}\right)}^{2}}{\left(d_{x,y}-r_{x,y}\right)}^{2}-E_{a}\\ -\frac{2E_{a}}{{\left(i_{x,y}-r_{x,y}\right)}^{2}}{\left(d_{x,y}-i_{x,y}\right)}^{2}\\ 0 \end{array}\right.\;\begin{array}{l} d_{x,y}<r_{x,y}\\ \begin{array}{l} r_{x,y}\leq d_{x,y}<\frac{i_{x,y}+r_{x,y}}{2}\\ \frac{i_{x,y}+r_{x,y}}{2}\leq d_{x,y}<i_{x,y}\\ else. \end{array} \end{array}$$

**Fig 2:**
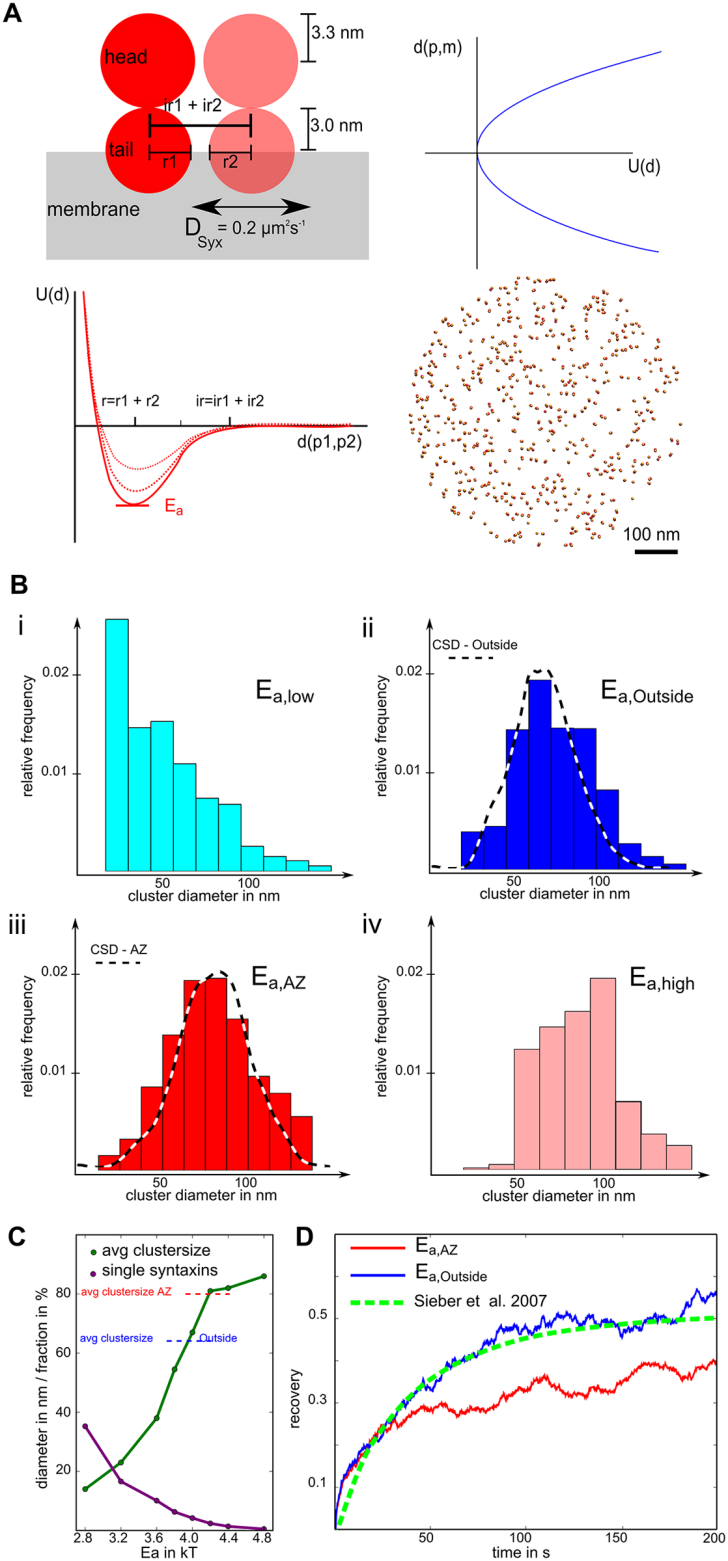
Computational model of Syntaxin-1A reproduces experimental results. (A) Schematic presentation of the computational model of Syntaxin-1A cluster formation. The ReaDDy two-particle model of Syntaxin (top left) with the “membrane” potential profile (top right) and the SNARE-SNARE attraction/clustering potential (bottom left) and the starting topology of 500 Syntaxins on a circular area with 300 nm radius (bottom right). (B) Cluster size distributions for different potential well depths (i-iv) show strong differences in the clustering behavior. The simulated cluster size distributions for the two potential parameters E_a,Outside_ (ii) and E_a,AZ_ (iii) correspond well to the experimental cluster size distributions found outside (CSD-Outside) and at active zones (CSD-AZ) shown as dashed lines in ii and iii, respectively. (C) Line-plot showing the average cluster size and the fraction of “single” syntaxins with respect to the potential strength parameter Ea, also indicated are average cluster size of active zone and outside region from the experimental STED data (dashed lines). (D) Recovery curves of simulated FRAP experiments for the two selected potential strengths compared with experimentally derived FRAP curve from Fig 4B in Sieber *et al*.[6].

Here, ***i***_***x*,*y***_ is the interaction radius between particles x and y. Two particles start to “feel” the clustering potential if their distance is below ***i***_***x*,*y***_, here chosen to be ***i***_***x*,*y***_ = ***r***_***x*,*y***_ + **2.25*nm***, which is the distance at which electrostatic interactions are expected to be negligible. With our settings, distances ***i***_***x*,*y***_ ≤ ***r***_***x*,*y***_ *+* **2.0*nm*** lead to almost no observed clustering. Larger distances do not have a strong effect on cluster sizes for ***E***_***a***_ **≤ 4.0*k***_***B***_ ***T***, however for ***E***_***a***_ *>* **4.0*k***_***B***_ ***T*** the longer range of the attractive potential counteracts the repulsion of the steric hindrance causing a cluster size limit, thus shifting the overall cluster size distribution towards larger clusters. The clustering potential was designed to resemble a potential of mean force for two membrane proteins [42] but at the same time simple enough to allow for efficient computation with a large timestep, resulting in a piecewise harmonic potential with a repulsive part and an attractive part. See Fig 2A for an illustration of the potential. The repulsive part reaches up to the collision radius of particles x and y (***r***_***x*,*y***_) and its strength is defined by the force constant ***k***_***r***_ which is chosen to be equal to ***E***_***a***_. The attractive part of the potential is active between ***r***_***x*,*y***_ and the interaction radius ***i***_***x*,*y***_ The parameter ***E***_***a***_ defines the well depth of the attraction potential. Since the actual interaction energy is not known and may vary for different lipid compositions and protein surroundings, ***E***_***a***_ is varied in order to find a value which can reproduce the cluster size distributions found in experiments.

For each tested value of ***E***_***a***_ = **{2.8,3.2,3.6,3.8,4.0,4.2,4.4,4.8}*K***_***B***_ ***T*** we have performed twelve simulations, each consisting of **50 · 10** ^**6**^ steps with a time step dt of 5 ns. Convergence of trajectories was tested by validating that no significant changes in the mean cluster size and cluster size distributions were observed. We then selected four values of ***E***_***a***_. Two values, ***E***_***a*,*outside***_ = **4*k***_***B***_ ***T*** and ***E***_***a*,*AZ***_ = **4.4 *k***_***B***_ ***T***, approximately reproduce the experimental cluster size distributions found outside and at active zones, respectively. Two additional values, ***E***_***a*,*low***_ = **3.6 *k***_***B***_ ***T*** and ***E***_***a*,*high***_ = **4.8 *k***_***B***_ ***T***, were chosen that exhibit more extreme cluster size distributions. For these setups we performed further simulations simply continuing from the endpoints of the convergence simulations, to analyze the equilibrium dynamics of syntaxin particles under different degrees of clustering. To study the aggregation time of syntaxin clusters at newly formed target sites such as synaptic vesicles approaching the presynaptic membrane, simulations were performed that also started from the converged configurations but with a static and highly attractive ***E***_***a***_ = **8*k***_***B***_ ***T*** syntaxin inserted to an empty membrane site.

Many analyses in this paper depend on the size of the syntaxin clusters. Here we define a cluster as a set of particles that are in proximity of at least one other particle in the set. Two particles are in proximity when they are not further than 1 nm apart from each other, i.e. when their centers are not further than ***r***_***x*,*y***_ + **1 *nm*** apart. In rare cases, this may lead to an overestimation of cluster sizes, since two close clusters might be joined to one larger cluster. However, we do not consider this to be a significant error. Firstly, because we average over many simulation steps and two distinct clusters will be clearly separated most of the time. Secondly, similar situation can arise for experimental data due to limited resolution making the simulated and experimental cluster size distributions comparable.

## Results

### Increased Syntaxin-1A cluster size within the active zone region of neuromuscular terminals

Chemical synapses release synaptic vesicles at specialized membranes (“active zones”), whose cytoplasmic faces are covered by electron dense structures reflecting the presence of extended molecular scaffolds. These cytoplasmic active zone scaffolds are meant to facilitate synaptic vesicle release by however so far largely unknown mechanisms. Importantly, scaffold sizes of individual active zones were found to scale with their microscopic release rates [43–45].

We here analyzed the Syntaxin-1A cluster distribution at glutamatergic *Drosophila* neuromuscular terminals of fixed larvae. In this preparation, active zones are found evenly spaced on the surface of nearly spherical boutons, easing analysis of active zone associated regulations. Active zone scaffolds are identified via labeling the ELKS/CAST family protein Bruchpilot (BRP). BRP, a large coiled-coil domain protein serves as a direct building block of the active zone cytomatrix [33,34,46,47]. Thus, to investigate the distribution of Syntaxin-1A clusters relative to active zone scaffolds, we used two-color STED microscopy to co-image Syntaxin-1A with Bruchpilot (Fig 1A).

In order to visualize BRP at the AZ, we previously used the monoclonal antibody NC82, which recognizes a small epitope at the very C-terminus of BRP. As by its monoclonal nature, the MAB NC82 governs only a low labeling density and thus is limited in visualizing the true extent of the active zone scaffold being present. In order overcome this issue, we produced a fusion protein antibody against the last 200 amino acids of BRP ("Last200"; contains the stretch recognized by Nc82 as well). Higher labeling density here gets obvious by denser, more extended labeling in STED images. BRP labeled with Last200 adopts a dense ring-like structure (top view) or a bar-like structure (lateral view) with a diameter of around 220-240nm (Fig 1A, column 1). The area enclosed by this ring plus a 25 nm padding was considered as an active zone. The remaining membrane area is considered as being “outside” of the active zone.

As already reported in previous studies, [24–29], we *per se* found Syntaxin-1A distributed over the entire presynaptic plasma membrane of NMJ terminals. However, the protein forms micro-domains or clusters as can be seen from the “patchy” structures in Fig 1A, column 2. On the level of individual active zones as dissected effectively by STED (Fig 1A, high magnifications in bottom row) Syntaxin-1A clusters appear to align well with the Bruchpilot ring structure and are often located in gaps of the active zone scaffold. Integrating the localization of Syntaxin clusters into a model of the architecture of active zones [48] it is apparent that the core active zone proteins, calcium channels, Rim-binding proteins and Bruchpilot are surrounded by four or five syntaxin-1A clusters which are adjacent but not right beneath the Bruchpilot C-terminus (Fig 1B, top view). This localization is consistent with the potential function of syntaxin clusters in docking, priming and fusion of SV's, which are known to dock at BRPc termini [34]. Quantitative analysis of Syntaxin-1A cluster localization revealed a relation between cluster size and position of Syntaxin (Fig 1C–1F). The analysis was performed for about 14 thousand clusters in 12 NMJs from 3 drosophila larvae. We used image analysis to determine the position and area of active zones, as well as the diameter of Syntaxin-1A clusters and their position in respect to these active zones. Active zones correspond to areas around and including the ring-like BRP structures. We compared Syntaxin-1A clusters that are situated within an active zone and those that are placed outside of an active zone. The cluster size distributions for both groups (Fig 1C) showed clearly that Syntaxin-1A clusters at active zones are on average larger than clusters outside of active zones, having an average diameter of 80nm compared to 64nm, respectively. To put this in perspective, the increase of the cluster diameter by 25% yields an increase of more than 50% in the number of Syntaxin molecules in the cluster. We quantified Syntaxin-1A cluster intensities and distances to active zones labeled by BRP C-term using image analysis (Fig 1D). The distance is here defined as the minimal distance of a Syntaxin-1A cluster to any BRP spot center, which is part of the BRP ring. Syntaxin-1A clusters close to BRP structures (< 100 nm distance) have a larger diameter (> 80 nm) than clusters that are at least 100 nm away from the next BRP structure (50–70 nm). Even differences of 50 nm in distance lead to significantly different mean cluster diameters, with p-values below 0.001. In addition, the fluorescence intensity of syntaxin-1A at active zones was around three times as high as outside of active zones (see Fig 1E for intensity distribution and also Kittel *et al*., 2006). This increase in fluorescence intensity can not only be explained by the increased Syntaxin-1A cluster size, but is mainly due to the fact that there are more syntaxin-1A clusters near active zones than outside. Fig 1F shows that the number of Syx-1A clusters per μm² at active zones is almost doubled compared to the outside area (Fig 1F). In summary, our data suggest that syntaxin-1A clusters are preferentially located and tend to be larger at active zones.

### Computational model of Syntaxin cluster formation

We used a simple computational model of syntaxin-1A populated membranes and performed particle-based diffusion simulations to investigate the formation, distribution and dynamics of Syntaxin-1A clusters. Syntaxin-1A was modeled as a dimer of two particles (Fig 2A). Particle 1 constraints syntaxin to the membrane and has an attractive interaction potential with other particles of type 1 (see Materials and Methods for details and Fig 2A for an illustration). The larger Particle 2 represents the bulky head domain of syntaxin and has a role in limiting the cluster size when syntaxins are packed, as suggested in [6]. We performed simulations of 500 randomly placed syntaxins on a 600 nm diameter disk with different parameter setups where only one parameter, the depth of the attraction potential *E*_a_, was changed. Fig 2B shows the cluster size distributions, each obtained from the average of twelve converged simulations for the respective parameter choices. Clearly, stronger interaction potentials favor the formation of clusters and increase the probability of observing large clusters. That is also emphasized by the correlation between the average cluster size and the fraction of single syntaxin molecules with the interaction energy shown in Fig 2C. The interaction energy values that best reproduced the Syntaxin-1A cluster size distributions observed in the STED image analyses are *E*_*a*,*outside*_ = 4*k*_*B*_ *T* and *E*_*a*,*AZ*_ = 4.4 *k*_*B*_ *T* for syntaxins outside or at the active zone, respectively. Note that these different parameter choices reflect an underlying molecular mechanism. A possible explanation for the increased syntaxin interaction energy at active zones is that interactions are also modulated by the phospholipid composition of the membrane which is supposed to be different at active zones [21]—however identification of the actual molecular mechanism is beyond the scope of the present study.

In order to validate the dynamics of our model, we simulated a FRAP (fluorescence recovery after photobleaching) experiment. Starting from equilibrated simulation frames, syntaxins in a disc-shaped area covering 10% of the full simulation area were marked as “bleached” while all other syntaxins were marked as “fluorescent”. The recovery of the fluorescent fraction as a result of the diffusional motion of syntaxins is then followed over time. Fig 2D shows these fluorescence recovery curves using both scenarios *E*_*a*,*outside*_ and *E*_*a*,*AZ*_ and confirms that “outside” syntaxins recover quantitatively similar to the FRAP measurements of [6] that were performed on PC-12 membrane sheets which do not contain active zones. Since most syntaxins are located in large clusters that move very slowly, the FRAP experiment not only probes the diffusion constant of syntaxin (which causes a recovery of about 15% during the first 5 s), but rather the association/dissociation dynamics to and from clusters (dominating the rest of the curve). Note that syntaxins at active zones recover more slowly, especially a much slower recovery phase is found after 30 s (Fig 2D). This difference at long timescales is expected due to the slower exchange dynamics for larger clusters. The similarity in recovery behavior for short timescales results from freely diffusing particles which behave equally in both cases and leading to a short phase of fast recovery.

In summary, the computational model employed here is able to reproduce the Syntaxin-1A cluster distribution and fluorescence recovery kinetics observed from experimental data. Besides the two interaction energy values chosen for active zones and outside regions (*E*_*a*,*outside*_ and *E*_*a*,*AZ*_), two additional interaction energies (*E*_*a*,*low*_ and *E*_*a*,*high*_) are considered in order to investigate the system behavior at more extreme conditions.

### Interplay between syntaxin clustering and exchange dynamics

**Fig 3:**
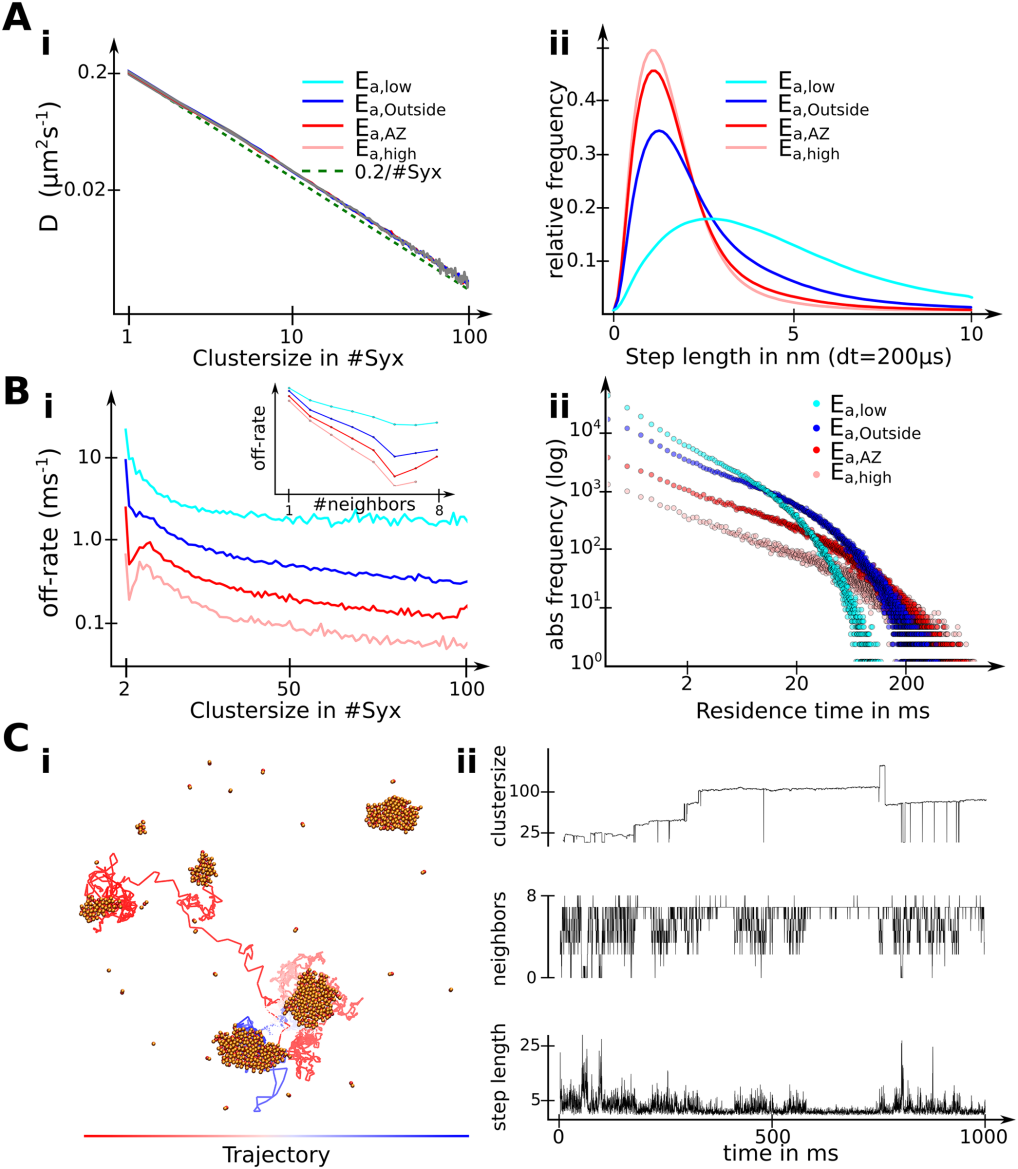
Degree of clustering influences Syntaxin-1A mobility and cluster dynamics. (A) Influence of clustering on Syntaxin-1A mobility. i) The diffusion constant of the cluster center is inversely proportional to the number of particles in the cluster. ii) The distribution of step lengths of single particles in a time period of 200 μs shows a wide variety of diffusive behaviors. Slow diffusion (small step length) is likely caused by particles in a clustered state, while faster diffusion (large step lengths) indicate to freely diffusing particles. (B) Cluster dynamics in relation to clustering degree. i) The rate of particles dissociating from a cluster as a function of the respective cluster size. ii) The distribution of residence times of particles in a cluster. (C) i) A sample trajectory of one Syntaxin-1A particle over the course of one simulation run. ii) Step length, number of neighbors and the size of the cluster in which the particle resides tracked for the sample trajectory.

Here, we will show how syntaxin clustering can affect SV docking and priming. For this, it is critical to understand the impact of the degree of clustering on the syntaxin-1A dynamics. To this end, we studied the diffusion of single syntaxin-1A particles and cluster, the syntaxin dissociation rate from clusters, and cluster life times. Simulations were performed for the four different parameters defined above, starting from already equilibrated configurations. Fig 3Ai shows the diffusion coefficient for clusters of different size. As expected, larger clusters diffuse much slower than small clusters or single Syntaxin-1A particles. In fact, the diffusion coefficient of clusters decreases linearly with the number of Syntaxin-1A particles in the cluster. The cluster diffusion coefficient itself is independent of the interaction energy *E*_*a*_, but as higher interaction energies are associated with increased numbers of large clusters, thus indirectly leading to slower diffusion in these cases. This strong interdependence of diffusion coefficient and cluster size effectively means that large clusters are nearly immobile while most of the dynamics are due to the fast motion of the syntaxin fraction that resides in small clusters. For example, it takes about ten seconds for a cluster with a 100 nm diameter to diffuse across an area equal to its own size, which is very long on the timescale relevant for exocytosis.

We next addressed diffusional mobility of individual syntaxin-1A particles/molecules. In Fig 3Aii, step lengths traveled within time intervals of 200 μs are shown. For a lower extend of clustering, syntaxin-1A particles showed a wide variety of step lengths, with many particles diffusing at speeds close to that of free diffusion of a single particle. For stronger clustering, however, the distribution of step lengths narrowed and shifted towards shorter step lengths that corresponded to the diffusion coefficients for medium-sized or even large clusters. Together with the findings from Fig 3Ai, this indicates that the clustering strength does not change the diffusion coefficient of single molecules or clusters, but rather the fraction of single molecules, small clusters and larger clusters. Given a short time dt, single or free particles have the same step length for weak and strong clustering but the particles in the weak clustering situation will be “free” more often.

Next, we investigated the off-rate and residence time of syntaxins in clusters as a function of cluster size and syntaxin interaction energy. Concerning cluster size, dissociation rates per particles decreased with increasing size of the cluster in which the particle resided (see Fig 3Bi). This can be explained by the strong dependence of the dissociation-rate on the number of neighbors of a particle (see Fig 3Bi inset). The dissociation rate decreases only mildly when going from medium-size clusters (20–40 syntaxins) to larger clusters. In such clusters most particles are “buried inside” the cluster and the number of dissociation-competent boundary particles grows only mildly with cluster size. Furthermore, the dissociation rate decreases significantly with increasing syntaxin interaction energy. Consequently, syntaxin clusters at active zones are longer-lived (as they are larger). Fig 3Bii presents an alternative view by plotting the residence time distribution of syntaxins as a function of syntaxin interaction energy. The higher syntaxin interaction energy at active zones is associated with a smaller fraction of particles that have a short lifetime. Still clusters inside and outside of active zones are rather dynamic as most particles can leave the cluster after a short period of time (< 2 ms) and only very few particles/molecules stay as long as our simulation period lasts (up to 1 s).

Note that the lifetimes of clusters could not be computed because above a critical size clusters grow large and then never disappear on any a timescale that can be practically simulated. In reality, it is possible that large clusters could disappear on timescales of minutes or longer, but this is probably a rare event. Clusters are still highly dynamic both in terms of their absolute size as well as in terms of their syntaxin composition, because individual syntaxins can still come and leave frequently.

Fig 3C illustrates the quantitative analyses taking a single but representative trajectory as an example. The life of a single syntaxin may be described as jumps between clusters. These jumps are time periods of free or almost free diffusion, associated with bursts of large step lengths and small cluster sizes / numbers of neighbors are observed. The jump sequence ends when the syntaxin molecule is absorbed by a cluster. Syntaxins are bound to small or large clusters for most of the time, but even then the particle is not fixed to a specific position but rather diffuses locally within the cluster, changing between the cluster boundary and the core. Individual syntaxin molecules are therefore neither free nor bound in nature, but rather dynamically exchange between free and clustered states continuously. Single particle tracking measurements of Syntaxin-1A at rat spinal cord neurons have also reported heterogeneous diffusion behavior, with slower diffusion and more pauses at active zones [30].

### Syntaxin-1A efficiently searches the membrane and can rapidly form clusters at docking sites

Syntaxin-1A at active zones has been suggested to mediate the docking, priming and fusion of synaptic vesicles [21]. Whether syntaxin-1A executes this role at membrane regions outside the active zone is less clear. Sites of vesicle docking outside of active zones were shown to exist and the importance of ectopic release has been hypothesized for some systems [49–52]. Here we investigate the potential physiological role of syntaxin-1A clusters at active zones, and the consequences of differences in cluster sizes and cluster dynamics observed inside and outside active zones.

**Fig 4:**
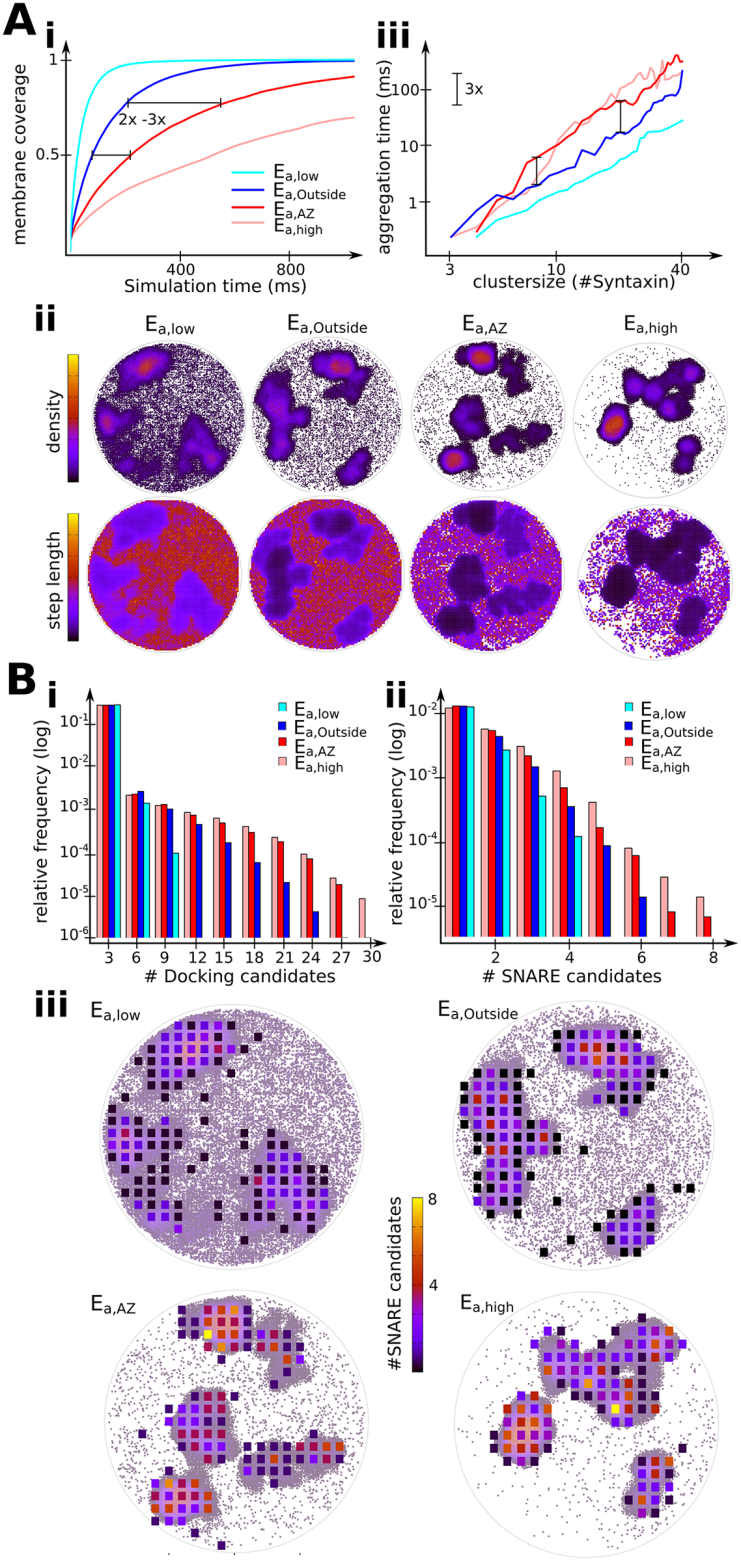
The differences in Syntaxin-1A mobility and cluster dynamics explain Syntaxin cluster function at specific locations. (A) Lower degree of clustering allows faster membrane exploration and aggregation at target sites. i) Progress of membrane coverage (percentage of visited area). ii) Density plots (upper row) and step length plots (lower row) of one sample simulation run for four different degrees of clustering. iii) The aggregation time of clusters at a newly formed target site as a function of cluster size. (B) Higher degree of clustering enables the formation of more SNARE complexes leading to higher fusion probability. i) Histogram of docking candidates, i.e. the number of all Syntaxin particles in a vesicle-sized area. ii) Histogram of SNARE candidates, i.e. the number of free Syntaxin particles and Syntaxins on the cluster rim. Candidates are tracked in an area with the size of a synaptic vesicle. iii) Positions of SNARE candidates superimposed on density plots. Areas with many SNARE candidates are at the periphery of clusters.

In order to efficiently dock vesicles outside active zones, but also to efficiently exchange syntaxins between different clusters, syntaxin must be able to rapidly explore the membrane surface. To determine the speed of exploration, the simulation area was divided into a lattice of vesicle-sized (diameter 40 nm) compartments and the fraction of compartments that are visited by at least one syntaxin molecule was tracked. The difference in membrane coverage between different degrees of clustering is considerable. The same area of membrane is covered up to three times faster outside of active zones than within an active zone (see Fig 4Ai), e.g. 50 percent of the simulation area is visited within 80ms outside in comparison to 225ms at the active zone, or 75 percent are visited within 200ms outside and 580ms at the active zone. However, in both scenarios the majority of the membrane surface can be explored within less than 1μs.

The existing syntaxin interaction energies are right at the balance between total aggregation and free diffusion as illustrated by the density plots of syntaxin-1A particles in Fig 4Aii. The plots show the syntaxin density integrated over one simulation. It is apparent that in all scenarios the large clusters remain localized and comprise the highest density. However, for interaction energies lower than *E*_*a*,*outside*_, even parts of the membrane without clusters are quite homogeneously covered, and the concentration of syntaxins in clusters is less pronounced. On the other hand, for interaction energies higher than *E*_*a*,*AZ*_, almost all syntaxins are bound to the large clusters and only a very small syntaxin population is mobile. A similar trend is apparent from the step length of particles. For low interaction energies, particle movement is similar all over the membrane, while stronger interaction energies almost abolish the movement within clusters and reduce the particle movement outside of clusters.

Thus, physiological Syntaxin-1A interaction energies seem adjusted in a way to rapidly explore the membrane and find synaptic vesicles. However, if a synaptic vesicle has been docked by a single syntaxin, will this event be able to recruit a cluster in a short time? To study this question, we simulated such a situation by introducing an attractive particle at a random position in the simulation area which is not yet occupied by syntaxin-1A. In Fig 4Aiii the aggregation time required to form clusters is shown as a function of cluster size. Clusters of 10 syntaxin molecules can form within a few milliseconds, while clusters of 40 syntaxins require about 100 milliseconds to form. Cluster aggregation takes longer (~3 times) with higher syntaxin interaction energies as found at active zones. This is due to the fact that higher interaction energies lead to slower exchange dynamics and thus fewer free syntaxins for the formation of new clusters. However, contrary to our setup, on a neuronal membrane syntaxins at active zone clusters can also be recruited from outside the active zone where there is a sufficient number of free syntaxins. Our simulation shows that syntaxin can efficiently find a dockable vesicle anywhere on the membrane and quickly form large syntaxin clusters at this position. These results are consistent with the cluster-granule colocalizations dynamics discussed in other studies [18,19].

### Implications of syntaxin clustering for vesicle docking and priming

We then turned to the physiological role of syntaxin-1A at active zones and investigated the effect of clustering on docking, priming and fusion of SVs. Docking is thought to be the initial contact formation between a synaptic vesicle and the plasma membrane, and believed to involve an interaction between syntaxin-1A/SNAP-25 with the vesicular protein synaptotagmin-1 [17]. Thus, any syntaxin in our simulation is considered to be docking competent. Next to Unc13 and Rim, priming involves the formation of a partially assembled SNARE complex between Syntaxin-1A, the vesicular Synaptobrevin, and SNAP-25 [53]. To this end, we evaluated the density of syntaxin molecules that were competent for either docking or priming. Since most of the important active zone proteins needed for priming are membrane-bound proteins, we suggest that a SNARE complex involving SNAP-25 can only be formed with accessible syntaxins that are either free or on the boundary—but not the inside—of a cluster, where other proteins bound to the membrane cannot enter. Furthermore, the timescale of priming is about 100 ms [54]. Therefore, syntaxins at cluster boundaries that stay in a localized region for at least 100 ms are called priming-competent.

In order to quantify our simulations for vesicle docking and priming, we divided the simulation area into a 20 × 20 lattice of compartments having areas of 30 × 30nm each, roughly corresponding to the area that would be accessible to vesicles close to the membrane surface. The number of docking- and priming-competent syntaxins within each such cell was counted, and the results reported in Fig 4B. The histograms in Fig 4Bi and 4Bii show that stronger clustering provides overall more candidates for both, vesicle docking and the formation of SNARE complexes. At active zones, there are about twice as many areas with 15 or more docking-competent syntaxins compared to outside. Also, active zones have sites with up to eight syntaxins that can form SNARE complexes compared to only up to four or six for weaker clustering outside of active zones. Fig 4Biii shows the spatial distribution of priming-competent SNARE complexes by superimposing the grid that was used for counting these candidates with the density plot (Fig 4Aii). In the same way as the density plot is more homogenous for weaker clustering affinities outside the active zones, the distribution of SNARE complex candidates is more diverse. This means that there are more areas where potentially one or two SNARE complexes can form but almost no areas with more than four potential SNARE complexes. At the active zone, however, we see fewer areas with priming-competent syntaxins, but most of these areas contain four to eight candidates for SNARE complex formation. All potential areas that are suitable for SNARE complex formation are located around sites of clustering, as has been suggested by Bar-On et al [23]. The areas with the highest number of candidates are at the immediate periphery of large clusters and therefore also close to a potential site for synaptic vesicle docking [4,20].

## Discussion

### Syntaxin is not uniformly distributed over the neuronal membrane

The function of proteins, in particular membrane proteins, strongly depends on their localization and distribution in the cell and its recruitment to specific sites [55–59].

Protein microdomains such as clusters with sizes in the range of 10 to 100 nm are smaller than the diffraction limit and can thus not be characterized by conventional light microscopy. With the advent of super-resolution microscopy techniques, more detailed investigation of protein distributions along the membrane are now possible [60–63]. The clustering of Syntaxin-1A has been well studied using super-resolution microscopy, both in terms of the size distribution and the co-localization with other SNARE proteins [6,23,64,65]. However, the distribution and clustering of Syntaxin-1A in relation to active zones, the sites at which the physiological neurotransmission takes place, has not yet been studied with high resolution imaging techniques. This is likely due to the fact that common model systems (e.g. PC-12 cells) do not contain active zones. A single molecule tracking study has reported changes in Syntaxin-1A dynamics at active zones [30] but did not relate to the existence, size and distribution of syntaxin-1A clusters.

Here we used super resolution light microscopy (STED) and image analysis to investigate the relation between Syntaxin-1A clustering and active zones. While Syntaxin-1A clusters are found all over the neuronal membrane, Syntaxin-1A clusters at active zones are significantly more abundant and on average larger in comparison to clusters outside of active zones.

Protein clusters on the membrane can arise from different mechanisms. It is known that Syntxin-1As are subject to weak homophilic interactions between their SNARE domains [5,6]. It is less clear which mechanism limits the size of clusters, i.e. prevents all syntaxin from merging into a single cluster. One contribution could be that splitting larger into smaller clusters is entropically favorable. Another likely contribution is the existence of bulky head domains in Syntaxin-1A that would disfavor the addition of syntaxins beyond a few tens to 100 by space exclusion, similar like in a “bouquet of flowers” [6]. This observation was represented in our dimer model of Syntaxin-1A, where the size of the larger particle in fact limits larger cluster sizes.

The actual Syntaxin-1A size distribution in our model is controlled by the magnitude of the syntaxin interaction energy *E*_*a*_. Here we modeled the different size distributions of syntaxin clusters inside and outside active zones by different *E*_*a*_ values. The molecular mechanism of this difference is still elusive. Clusters may be induced by differences in lipid composition such as lipid rafts [66,67] or PiP2 islands [14,68]. Thus, differences in clustering behavior may be induced by differences in PiP-composition inside and outside of the active zone, which may result from active-zone associated PiP kinases or phosphatases. PiP3 and PiP2 have an increased concentration at active zones and have been shown to increase clustering of Syntaxin by changing the interaction between Syntaxins, therefore mediating cluster formation [14,15].

Protein clusters may also be induced by membrane proteins or protein skeletons on the surface of membranes [67,69,70]. Thus, syntaxin clusters could be attracted to active zones via favorable interactions with other active zone proteins [8,30,65,71].

### Physiological role of syntaxin clusters

A number of functions have been attributed to syntaxin clusters [21]. Clusters can act as buffers preventing the formation of “unproductive” complexes with other SNARE proteins [61,72] by shielding the SNARE domain of Syntaxin-1A, allowing to put a high concentration of syntaxins right at the site where they are needed. Clustering of Syntaxin-1A might also facilitate efficient recycling of cis-SNARE complexes after fusion allowing a faster restoring of the fusion site [73]. Furthermore, there is evidence that Syntaxin clusters act as docking and fusion sites for vesicles [16,18].

Docking of vesicles likely involves the contact formation of Syntaxin and vesicular Synaptotagmin [17] and precedes the priming and fusion of vesicles. Thus, syntaxin clusters might facilitate vesicle docking because the number of Syntaxins that are simultaneously accessible to a vesicle near the membrane is higher at a cluster site compared to freely diffusing syntaxin. Subsequently to docking, the priming of vesicles involves the formation of ternary SNARE complexes, consisting of syntaxin, synaptobrevin and SNAP-25. It is known that efficient fusion requires *in vivo* at least three SNARE complexes [74], such that increasing the number of locally available syntaxins competent to form ternary complexes is important for priming and fusion efficiency [75–77]. A high density of such syntaxin molecules is available at the boundaries of syntaxin clusters. Thus, syntaxin clusters may be platforms for docking vesicles “on top” and priming/fusing vesicles “next to” them [23].

Here we quantified the number of docking and priming-competent Syntaxin-1A molecules that a vesicle close to the membrane would see. These numbers are greatly increased at clusters compared to regions outside clusters, or in a situation where no syntaxin clustering occurs at all. Both numbers are further increased at active zone, where syntaxin clusters are more frequent and larger. The differences in syntaxin cluster size and density are not the sole determinant of docking and fusion behavior, but combined with the fact that synaptic vesicles are tethered at active zone proteins [34,78] this contributes to the efficiency of docking and priming at active zones compared to other regions of the synaptic membrane.

To identify the actual contribution of syntaxin clustering, one would have to compare the number of docked vesicles and fusion events to a system with lower clustering at active zones. The mutation of specific amino acids near the transmembrane region of syntaxin [13] could provide for such a system. This system could be used for high pressure freeze (HPF) and subsequent EM analyses to investigate a possible reduction of the docked synaptic vesicle pool. Furthermore, such mutations that interfere with Syntaxin1 clustering might de-/increase the release and give a first idea about their functional contribution.

### Importance of weak interactions and cluster dynamics

If syntaxin clusters are important for neuronal function, then the dissociation of syntaxin from clusters, the mobility of syntaxins and clusters on the membrane, and the replenishment of clusters are equally important during the cycle of vesicle exo-/endocytosis. Computational models integrating experimental data on syntaxin cluster localization and diffusion [6,23,73] have brought interesting insights about the dynamics of syntaxin clusters. The computational model of syntaxin cluster formation introduced in this work is able to reproduce both, the cluster size distributions obtained from imaging analyses and the diffusion behavior as seen in [6]. The model is used to predict the dynamics of syntaxin clusters, such as dissociation rate and aggregation speed. We find that the dissociation rate reduces with increasing cluster size, resulting in longevity of large clusters. Moreover, larger clusters are extremely slow, although syntaxins are still able to diffuse inside the cluster and thus exchange between boundary and core positions. The mobility of syntaxins arises from the ability to dissociate from a cluster, and then cover a large distance in a short time by diffusing between the clusters. In this way, the system remains extremely mobile and is able to “search” the entire synaptic membrane efficiently, even though most of the syntaxin molecules are associated to largely immobile clusters most of their lifetime.

Interestingly, increasing the syntaxin interaction energy only by a fraction of 1 *k*_*B*_ *T* leads to stronger clustering at the expense of losing the free and mobile syntaxin fraction. Vice versa, slightly decreasing the syntaxin interaction energy results in losing large clusters. It thus appears that syntaxin at neuronal membranes is set up in a way to strike a balance between having most syntaxins aggregated in clusters while at the same time maintaining the ability for small fraction of free syntaxins to rapidly search the membrane surface. This balance can be shifted by subtle effects that slightly enhance or decrease the syntaxin interaction. At active zones, the system is pushed towards its upper limited of cluster aggregation while still keeping a small mobile fraction, and thus optimized for vesicle docking, priming and fusion. Outside active zones, the system is pushed towards the lower limit of cluster aggregation and optimized for mobility.

### Dynamical model of the role of syntaxin clustering in exocytosis

We summarize our insights in the following model for the function of syntaxin clusters at active zones and outer regions. The large and stable clusters at the active zone represent potential sites for vesicle docking through its high local concentration of interaction partners of vesicular proteins. The periphery of these large clusters shows a high probability for SNARE complex formation, which also implies a higher fusion probability [74–77]. Together with the recycling capabilities of syntaxin clusters [73] and their role as reserve pool [61], this makes these regions the ideal SV fusion sites. These features contribute to active zones being regions of high synaptic vesicle fusion activity. Yet stronger clustering at the active zone leading to even larger and more stable clusters would provide only a small increase in syntaxin candidates for SNARE complex formation but would reduce the accessibility of syntaxin by fixing them to clusters. Thus, syntaxins at the active zone may operate at an upper limit of aggregation while still permitting dynamics.

The smaller and more dynamic syntaxin clusters outside the active zone might act as a flexible reserve pool of syntaxins for clusters at the active zone and might also allow for ectopic release by spontaneous aggregation and docking of vesicles approaching parts of the membrane outside of active zones. A stronger clustering at these outer regions would impair these functions. Furthermore, the difference in syntaxin interaction energies inside and outside active zones explains the much higher density of syntaxin at active zones. The more attractive clusters at the active zone act as syntaxin sinks that recruit syntaxin molecules from the outside into the active zone. Therefore, having a difference between the syntaxin interaction energies inside and outside active zones is important. A more detailed analysis of the impact of syntaxin clustering on exocytosis could be achieved by investigating interactions with other proteins involved in exocytosis, e.g. Unc-13, through colocalization studies or electrophysiology experiments. This would allow for a more sophisticated computational model including reactions leading to synaptic vesicle fusion. Such reaction models [23,79] can be easily integrated in the existing modeling framework.

The clustering of membrane proteins via weak protein-protein interactions is a fine tuned process as small changes in the protein-protein interaction can have a strong effect on the proteins dynamic behavior. Such a self-organized clustering not only can establish a specific spatial distribution of membrane proteins but can also provide an elegant way to yield location-specific functionality of these proteins and their clusters. The formation of protein clusters through weak protein-protein or protein-lipid interactions has been observed for the other SNAREs [4,23], other exocytotic proteins such as synaptotagmin [80,81], and for several other systems such as membrane receptors [82,83], where the biological role of their clustering is also debated [55,57]. Therefore, a study of the nature of such interactions and the resulting dynamic behavior will also be relevant for the study of other system than syntaxin [56,63,84–86].
